# Effect of Growth Rate on the Crystal Orientation and Magnetization Performance of Cobalt Nanocrystal Arrays Electrodeposited from Aqueous Solution

**DOI:** 10.3390/nano8080566

**Published:** 2018-07-24

**Authors:** Ryusei Saeki, Takeshi Ohgai

**Affiliations:** 1 Graduate School of Engineering, Nagasaki University, Bunkyo-machi 1-14, Nagasaki 852-8521, Japan; bb52318101@ms.nagasaki-u.ac.jp; 2 Faculty of Engineering, Nagasaki University, Bunkyo-machi 1-14, Nagasaki 852-8521, Japan

**Keywords:** cobalt, nanocrystal, nanochannel, electrodeposition, magnetization

## Abstract

The formation work of a two-dimensional hcp-Co (metallic cobalt crystal with hexagonal close packed structure) nucleus, *W_hkl_*, was calculated by Pangarov’s theory. *W*_002_ was estimated to be smaller than *W*_100_ in a cathode potential range nobler than the transition potential, *E*^tra^ (ca. −0.77 V vs. Ag/AgCl). To confirm the above estimation, ferromagnetic nanocomposite thick films, which contained (002) textured hcp-Co nanocrystal arrays, were synthesized by potentiostatic electrochemical reduction of Co^2+^ ions in anodized aluminum oxide (AAO) nanochannel films with ca. 45 µm thickness. The aspect ratio of hcp-Co nanocrystals with a diameter of ca. 25 nm reached up to ca. 1800. Our experimental results revealed that the texture coefficient, *TC*_002_, increased when decreasing the overpotential for hcp-Co electrodeposition by shifting the cathode potential nobler than *E*^tra^. In a similar way, *TC*_002_ increased sharply by decreasing the growth rate of the hcp-Co nanocrystals so that it was smaller than the transition growth rate, *R*^tra^ (ca. 600 nm s^−1^). The perpendicular magnetization performance was observed in AAO nanocomposite films containing hcp-Co nanocrystal arrays. With increasing *TC*_002_, the coercivity of the nanocomposite film increased and reached up to 1.66 kOe, with a squareness of ca. 0.9 at room temperature.

## 1. Introduction

The Curie temperature of metallic cobalt is 1065 °C, which is the highest of all materials [1]. Hence, when a magnet is used under high temperature conditions, cobalt-based metallic materials are the most promising, compared to cobalt-containing oxide materials [2]. The metallic cobalt crystal with hexagonal close packed structure (hcp-Co) is spontaneously magnetized along to the c-axis direction (002), similar to the ferroelectric behavior of BaTiO_3_ with a hexagonal structure [3]. This hexagonal structure induces a large magneto crystalline anisotropy energy in hcp-Co crystals [4,5]. Therefore, hcp-Co with one dimensional structure is the best candidate for the hard-magnetic materials used under high temperature conditions [6]. In particular, cobalt alloy nanocrystal arrays with a high aspect ratio shows novel physical and chemical performances, such as electron transportation, anisotropic magnetization, and electrochemical catalytic reactions [7]. Concerning the fabrication method of cobalt nanocrystal arrays, the template-based method—which uses nanochannel structures, such as ion track-etched foils or anodized aluminum oxide (AAO) membranes [8,9,10,11,12]—is a promising technique which enables us to realize an ideal straight shape of nanocrystals with high density arrays and a high aspect ratio. Specifically, the template-based method using AAO nanochannel templates has many advantages compared with other techniques including mechanical strength, heat resistance, and cost performance.

Some researchers have reported that cobalt nanocrystal arrays can be electrodeposited in nanochannel templates and exhibit some characteristic magnetic properties [13,14,15]. Garcia et al. reported that radially distributed cobalt (Co), nickel (Ni), and Co-Ni alloy nanocrystal arrays were able to be electrodeposited in nanochannels of an AAO layer on the surface of a metallic aluminum rod by using a pulsed current electrodeposition technique [16,17]. They revealed that squareness and coercivity of the radially distributed Co nanocrystal arrays (average diameter of 35 nm, average length of 2.5 µm, and average aspect ratio of ca. 70) were 0.44 and 1.2 kOe, respectively. Samardak et al. also reported that compositionally tuned Co-Ni alloy nanocrystal arrays were able to be electrodeposited in nanochannels of an AAO layer on the surface of a metallic aluminum foil by using an alternating current (AC) electrodeposition technique [18]. They found that squareness and coercivity of the electrodeposited Co alloy nanocrystal arrays (average diameter of 30 nm, average length of 3 µm, and average aspect ratio of ca. 100) were 0.52 and 0.86 kOe, respectively. Ghemes et al. also reported that Co_35_Fe_65_ alloy nanocrystal arrays can be prepared using a pulsed potential deposition technique [19]. They revealed that squareness and coercivity of the Co alloy nanocrystal arrays (average diameter of 35 nm, average length of 15 µm, and average aspect ratio of ca. 430) were 0.5 and 0.8 kOe, respectively.

It is well known that the magnetic force of permanent magnetic materials increases with an increase in the surface magnetic flux density. The magnitude of the surface magnetic flux density depends on the volume of the magnetic materials, while the magnetic coercivity of permanent magnetic materials decreases with an increase in the crystal grain size of the magnetic materials. Hence, if magnetic nanocrystal arrays are applied for permanent magnetic materials with an anisotropic magnetization performance, an industrial production line will require magnetic nanocrystals with (002) textured structure, high density array, and high aspect ratio. However, in the previous works, the aspect ratio of cobalt nanocrystals with a (002) textured structure did not reach 1000. Recently, we reported that iron (Fe) nanocrystals with an aspect ratio of 2000 can be electrodeposited by a pulsed potential deposition technique using AAO nanochannels with a diameter of ca. 30 nm [20]. It is well known that the AAO nanochannel’s diameter size decreases by decreasing the anodization voltage and solution temperature. However, in our previous study, it was found that a minimum anodization voltage of 30 V was required to maintain an AAO nanochannel structure with a large aspect ratio when an aqueous solution containing oxalic acid was used as an electrolytic solution for the anodization process [20]. Masuda et al. reported that an AAO nanochannel structure with an aspect ratio of several thousands was realized at an anodization voltage of 25 V when an aqueous solution containing sulfuric acid was used [21]. Hence, in this study, we tried to fabricate AAO nanochannel templates with an aspect ratio of ca. 2000 at an anodization voltage of 20 V by using an aqueous solution containing sulfuric acid under low temperature conditions (less than 2 °C). Then, we calculated the formation work of a two-dimensional hcp-Co nucleus, *W*_hkl_, by Pangarov’s theory [22,23,24,25] to determine the transition potential, *E*^tra^, which enables hcp-Co crystals to have a (002) preferential orientation. After that, using the obtained AAO nanochannel templates, we tried to synthesize (002) textured hcp-Co nanocrystal arrays by using a potentio-static electrodeposition technique to confirm the above estimation of *E*^tra^ and to realize an anisotropic magnetization performance with large squareness and coercivity.

## 2. Materials and Methods 

**Figure 1:**
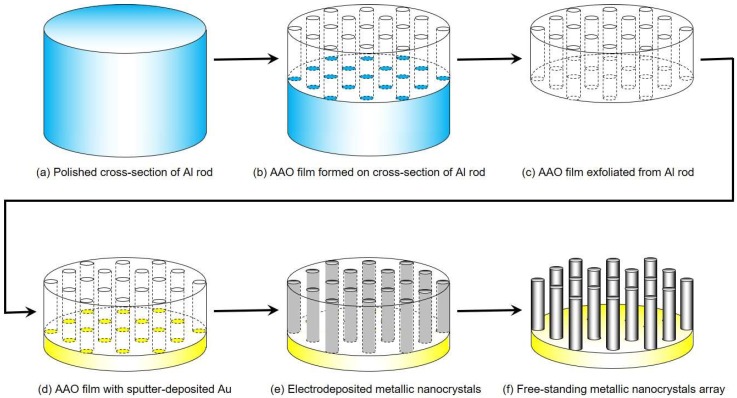
Fabrication process of free-standing metallic nanocrystal array. (**a**) Polished cross-section of Al rod, (**b**) anodized aluminum oxide (AAO) film formed on the cross-section of Al rod, (**c**) AAO film exfoliated from Al rod, (**d**) AAO film with sputter-deposited Au, (**e**) electrodeposited metallic nanocrystals, and (**f**) free-standing metallic nanocrystals array.

First, a cross-section of a pure aluminum rod (99.5%) with a diameter of 10 mm was mechanically polished by an automatic grinder. Subsequently, the cross-section of the aluminum rod was electrochemically polished in an ethanol solution containing perchloric acid to obtain a mirrorlike surface, as shown in Figure 1a. Next, anodization was conducted by applying a constant cell voltage of 20 V in 0.3 mol·L^−1^ sulfuric acid at 2 °C for 24 h to form an AAO nanochannel layer (ca. 45 µm in thickness) on the polished cross-section of the aluminum rod, as shown in Figure 1b. Then, the AAO nanochannel layer was exfoliated from the aluminum rod by applying an anodization voltage of 35 V for 30 s in an ethanol solution containing perchloric acid, as shown in Figure 1c. Prior to electrodeposition, a thin gold layer (ca. 200 nm in thickness) was sputter-deposited on one side of the AAO nanochannels, as shown in Figure 1d. The cobalt deposition was conducted in an aqueous solution containing 0.5 mol·L^−1^ cobalt chloride hexahydrate and 0.4 mol·L^−1^ boric acid at a temperature of 80 °C. The AAO nanochannels with a thin gold layer served as a cathode, a gold wire was used as an anode, and a silver wire with silver chloride in a saturated aqueous solution of potassium chloride (Ag/AgCl) was used as a reference electrode. Cobalt nanocrystals were potentio-statically electrodeposited in the AAO nanochannels, as shown in Figure 1e. During the electrodeposition, the cathode potential was fixed to −0.65 V (ca. 320 s), −0.70 V (ca. 260 s), −0.75 V (ca. 150 s), −0.80 V (ca. 100 s), and −0.85 V (ca. 80 s). After the electrodeposition, the AAO nanochannel layer was dissolved in 5 mol·L^−1^ NaOH aqueous solution to obtain a free-standing cobalt nanocrystal array as shown in Figure 1f.

Structure and crystallographic orientation of the cobalt nanocrystal arrays were characterized by a scanning electron microscope (SEM, JSM-7500FA, JEOL Ltd., Tokyo, Japan) and an X-ray diffractometer (XRD, Miniflex600-DX, Rigaku Corp., Tokyo, Japan). Magnetic properties of the cobalt nanocrystal arrays were investigated using a vibrating sample magnetometer (VSM, TM-VSM1014-CRO, Tamakawa Corp. Ltd., Sendai, Japan) at room temperature. The hysteresis loops were obtained in the magnetic field which was applied along the perpendicular and in-plane directions, with external magnetic fields up to 10 kOe. The perpendicular direction corresponds to the long axis of the cobalt nanocrystals, which is perpendicular to the plane of the AAO nanochannel film, while the in-plane direction corresponds to the short axis of cobalt nanocrystals, which was in-plane with the AAO nanochannel film.

## 3. Formation Work of a Two-Dimensional hcp-Co Nucleus Based on Pangarov’s Theory

Pangarov et al. reported that the preferential crystalline orientation could be estimated by calculations using two-dimensional nuclei theory [22,23,24,25]. According to their reports, the work of formation of two-dimensional nuclei, *W_hkl_*, for the various lattice planes is given by the following (Equation (1)).

(1) $$W_{hkl}=\frac{B_{hkl}}{\frac{1}{mN}\left(\mu -\mu_{0}\right)-A_{hkl}},$$

where, *m* is the number of atoms in the gas molecule and *N* is Avogadro’s number. Also, *μ* denotes the chemical potential of the equilibrium vapor above the two-dimensional nuclei and *μ*_0_ denotes the chemical potential of the vapor at equilibrium with an infinitely large three-dimensional crystal. *A_hkl_* is the difference between separation work (*φ*^0^) of one atom from a three-dimensional crystal and separation work (*φ*^0^*_hkl_*) of one atom from a two-dimensional crystal. *B_hkl_* is the parameter concerning the specific edge energy [25].

In the case of electrodeposition of metals, Equation (1) above may be written as the following (Equation (2)).

(2) $$W_{hkl}=\frac{B_{hkl}}{\frac{zF}{N}\left(E-E^{eq}\right)-A_{hkl}},$$

where, *z* is the valence and *F* is Faraday constant. Also, *E* denotes the electrode potential at the respective current density, and *E^eq^* denotes the equilibrium electrode potential.

If (002) orientation occurs preferentially, *A_hkl_* and *B_hkl_* are given by the following equations.

(3) $$\begin{array}{c} A_{002}=\varphi^{0}-\varphi_{002}^{0}=\left(6\psi_{1}+3\psi_{2}+\psi_{3}+9\psi_{4}\right)-\left(3\psi_{1}+3\psi_{4}+\psi_{u002}+\psi_{s}\right)\\ =3\psi_{1}+3\psi_{2}+\psi_{3}+6\psi_{4}-\psi_{u002}-\psi_{s} \end{array}$$

(4) $$B_{002}=3\psi_{1}^{2}+\frac{9}{2}\psi_{1}\psi_{4},$$

If (100) orientation occurs preferentially, *A_hkl_* and *B_hkl_* are given by the following equations.

(5) $$\begin{array}{c} A_{100}=\varphi^{0}-\varphi_{100}^{0}=\left(6\psi_{1}+3\psi_{2}+\psi_{3}+9\psi_{4}\right)-\left(\psi_{1}+\psi_{3}+\psi_{u100}+\psi_{s}\right)\\ =5\psi_{1}+3\psi_{2}+9\psi_{4}-\psi_{u100}-\psi_{s}, \end{array}$$

(6) $$B_{100}=\psi_{1}\psi_{3},$$

where, *φ*^0^ is the work of separation of one atom from a half-crystal position for the hcp lattice in the case of a three-dimensional crystal, and *φ*^0^*_hkl_* is the work of separation of one atom from a half-crystal position for the (*hkl*) lattice plane in the case of a two-dimensional crystal. Parameters, *ψ*_1_, *ψ*_2_, *ψ*_3_ and *ψ*_4_ are the work for breaking a bond between first, second, third and fourth neighbors, respectively. Also, *ψ*_u_ denotes the work for separating one atom from under the metallic layer and *ψ*_s_ denotes the work for separating one atom from the surface ionic layer. Based on Lennard-Jones potential model [26] and the bonding energy of Co (383 kJ·mol^−1^) [27], *ψ*_1_, *ψ*_2_, *ψ*_3_ and *ψ*_4_ are estimated to be 9.41 × 10^−20^ J, 1.18 × 10^−20^ J, 4.96 × 10^−21^ J and 3.49 × 10^−21^ J, respectively. Also, *ψ*_u002_, *ψ*_u100_ and *ψ*_s_ are estimated to be 1.72 × 10^−19^ J, 2.69 × 10^−19^ J and 1.74 × 10^−19^ J, respectively. Hence, *A*_002_, *B*_002_, *A*_100_ and *B*_100_ are determined to be −0.02 × 10^−19^ J, 2.80 × 10^−38^ J^2^, 0.94 × 10^−19^ J and 4.67 × 10^−40^ J^2^, respectively. 

**Figure 2:**
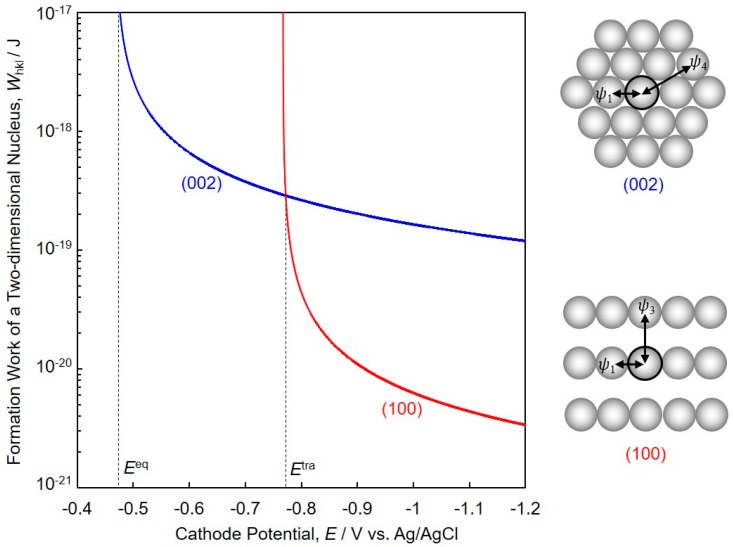
Theoretical calculation of the relationship between formation work of a two-dimensional nucleus, *W_hkl_*, and cathode potential, *E*, for the electrodeposition of hcp-Co (metallic cobalt crystal with hexagonal close packed structure) with (002) or (100) preferential orientation.

Figure 2 shows theoretical estimation concerning the formation work of a two-dimensional nucleus, *W_hkl_* calculated by using equation (2). This figure reveals the relationship between *W_hkl_* and cathode potential, *E* for the electrodeposition of hcp-Co with (002) or (100) preferential orientation. According to Figure 2, *W*_100_ is smaller than *W*_002_ when the cathode potential range is less noble than the transition potential, *E*^tra^ (ca. −0.77 V vs. Ag/AgCl). Hence, (100) orientation will occur preferentially in a potential range that is less noble than *E*^tra^. On the other hand, *W*_002_ is smaller than *W*_100_ when the cathode potential range is nobler than *E*^tra^. Therefore, (002) orientation will occur preferentially in a potential range that is nobler than *E*^tra^.

## 4. Results and Discussion

### 4.1. Fabrication of AAO Nanochannel Templates

**Figure 3:**
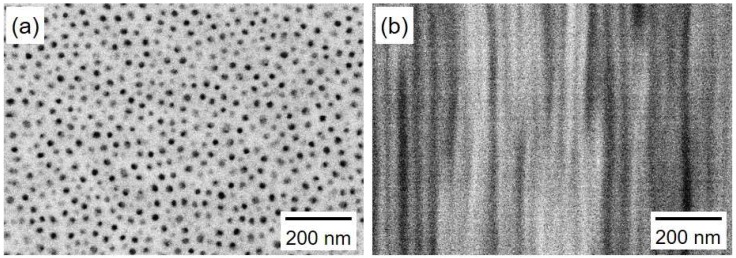
Planar-view (**a**) and cross-sectional (**b**) SEM images of anodized aluminum oxide nanochannel templates that were anodized at 20 V for 24 h. The electrolytic solution temperature was kept at 2 °C during the anodization.

Figure 3 shows SEM images of a planar view (a) and a cross-section (b) of an AAO nanochannel film. The pores have a nanochannel structure with a through-hole, dense-packed, and parallel-aligned geometry without branches. The obtained pore diameter *D_P_*, inter-pore distance *D_i_*, and pore length *L* from the SEM images resulted in an average value of ca. 25 nm, ca. 50 nm, and ca. 45 µm, respectively. Hence, the aspect ratio of the nanochannel is estimated to be ca. 1800.

### 4.2. Electrodeposition of Cobalt Nanocrystal Arrays

**Figure 4:**
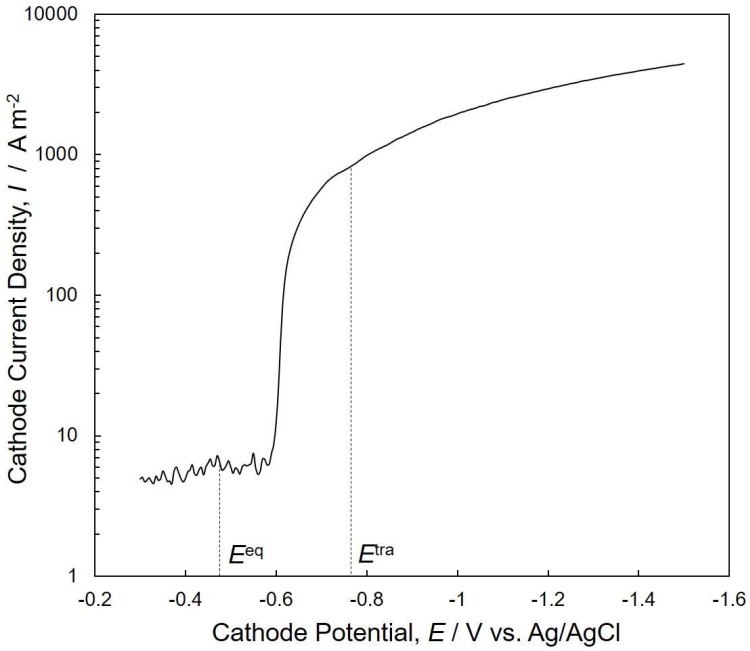
Cathode polarization curve for Co electrodeposition from an aqueous solution containing CoCl_2_ and H_3_BO_3_. The solution temperature was kept at 80 °C.

Figure 4 shows a cathodic polarization curve for cobalt deposition on a metallic copper sheet. The cathode potential was linearly scanned from −0.30 V to −1.50 V at a rate of 50 mV·s^−1^. Constant small current densities of approximately 5 A·m^−2^ were measured from −0.30 V to −0.58 V. The equilibrium potential of Co/Co^2+^ in the experimental conditions was estimated to be ca. −0.48 V vs. Ag/AgCl according to Nernst’s Equation, as shown in the following (Equation (7)).

(7) $$E^{eq}=E^{0}+\frac{RT}{zF}ln\frac{\left[M^{z+}\right]}{\left[M^{0}\right]},$$

where, *E*^0^ = −0.27 V vs. Ag/AgCl, *R* = 8.3 J K^−1^·mol^−1^, *T* = 353 K, *z* = 2, *F* = 96485 C·mol^−1^, and [*M^z^*^+^]/[*M*^0^] = 0.5. Therefore, the observed constant small current in the potential, ranging from −0.30 V to −0.58 V, is mainly attributed to hydrogen evolution, which usually takes place as a competitive reaction with metal deposition in aqueous solution [28].

A sharp increase was observed at the potential ranging from −0.58 V to −0.65 V. This sharp increase of current corresponds to the start of Co deposition, considering the equilibrium potential of Co/Co^2+^ (−0.48 V vs. Ag/AgCl). In the potential range from −0.65 V to −1.50 V, the slope of the curve decreased with increasing cathodic overpotential. It is well known that the optimum cathode potential range for Co deposition can be determined by a cathodic polarization curve obtained in a wide cathode potential regime [29]. Considering the result obtained from Figure 4, the optimum cathode potential range for Co deposition was determined to be less than −0.65 V.

**Figure 5:**
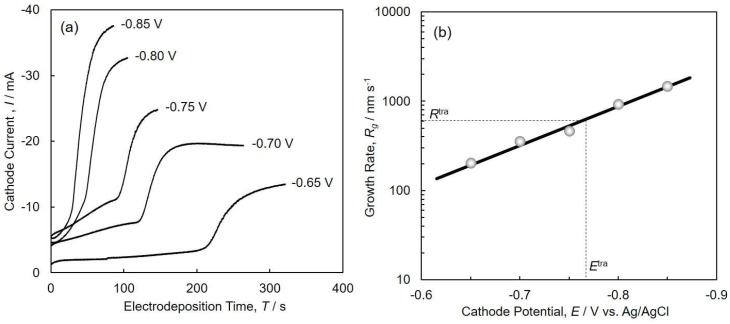
(**a**) Time-dependence of cathode current during the electrodeposition of Co nanocrystals in the nanochannels of anodized aluminum oxide templates. (**b**) Effect of cathode potential on the growth rate of electrodeposited Co nanocrystal arrays.

Figure 5a shows the time-dependence of cathode current during the electrodeposition of Co nanocrystals in the nanochannels of the anodized aluminum oxide templates. At the initial stage, the cathode current showed an almost steady value because of the stable supply of cations from the bulk of solution to the pores [30]. In general, the end of the nanocrystal growth is reflected by a rapid increase in the current density because of cap growth on top of the templates. This is simultaneously accompanied by a continuous increase in the electrode area [31]. In our experiments, we utilized a membrane thickness of ca. 45 µm, which is the same as the lengths of the Co nanocrystals. For example, at the cathode potential of −0.75 V, the filling time (the time difference between the start and the sudden increase in the cathode current, according to Figure 5a) was ca. 100 s, and the growth rate was estimated to be ca. 500 nm·s^−1^.

Figure 5b shows the effect of cathode potential on the growth rate of electrodeposited Co nanocrystal arrays. The growth rate increases with increasing cathodic overpotential, and this slope corresponds well to the result obtained from Figure 4 in the potential range from −0.65 V to −0.85 V. At the transition potential, *E*^tra^ (ca. −0.77 V vs. Ag/AgCl), the transition growth rate, *R*^tra^, of the electrodeposited Co nanocrystal arrays was estimated to be ca. 600 nm s^−1^, as shown in Figure 5b.

**Figure 6:**
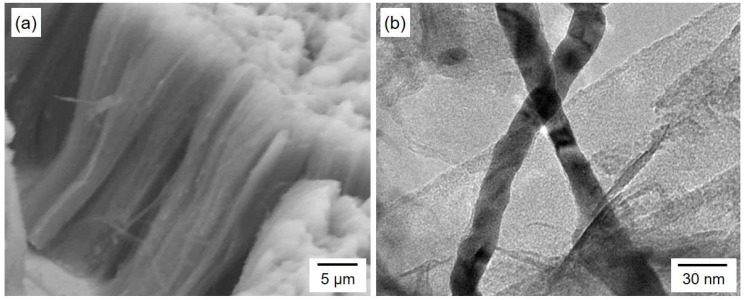
SEM image (**a**) and TEM image (**b**) of Co nanocrystals electrodeposited potentiostatically. Co nanocrystals were separated from an anodized aluminum oxide nanochannel template.

Figure 6 shows an SEM image (a) and TEM image (b) of Co nanocrystals electrodeposited potentiostatically. Co nanocrystals were separated from the anodized aluminum oxide nanochannel template. The one-dimensional structures were densely packed, and each nanocrystal lay in a parallel direction. The diameter of the Co nanocrystals was estimated to be ca. 25 nm. Considering a membrane thickness of ca. 45 µm, an ultrahigh aspect ratio of 1800 was achieved in our experiment. As shown in Figure 5a, Co nanocrystals, which were electrodeposited at the cathode potential ranging from −0.65 V to −0.85 V, reached the surface of the AAO templates. Hence, the average aspect ratio of Co nanocrystals, which were electrodeposited at the cathode potential range, was also ca. 1800.

### 4.3. Crystallographic Orientation of Co Nanocrystal Arrays

**Figure 7:**
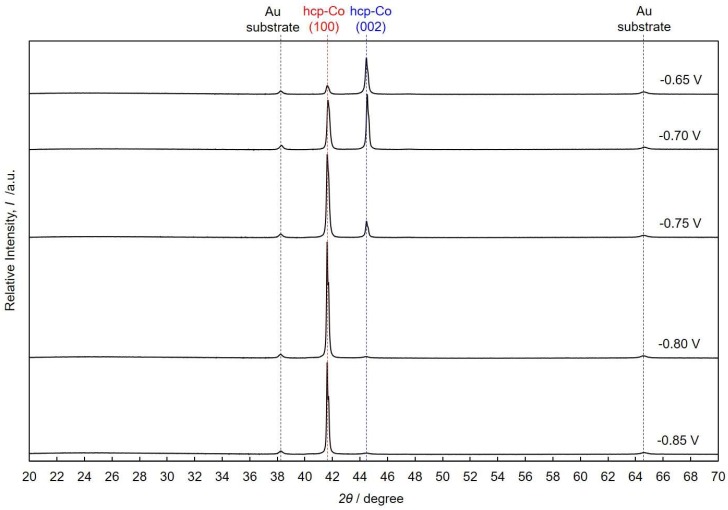
X-ray diffraction patterns of Co nanocrystal arrays that were electrodeposited at cathode potentials of −0.85 V, −0.80 V, −0.75 V, −0.70 V, and −0.65 V.

Figure 7 shows the X-ray diffraction patterns of Co nanocrystal arrays that were electrodeposited at cathode potentials of −0.85 V, −0.80 V, −0.75 V, −0.70 V and −0.65 V. Two peaks were observed at 2*θ* angles of 41.6° and 44.5°, which correspond to (100) and (002) of the Miller indices in hcp-Co crystal planes, respectively. In the hcp-Co nanocrystals that were electrodeposited at −0.85 V, (100) preferential orientation was observed. By decreasing the overpotential and shifting the cathode potential to a nobler direction, the peak intensity for (100) decreased and the one for (002) increased. On the contrary, in the hcp-Co nanocrystals that were electrodeposited at −0.65 V, (002) preferential orientation was observed, as shown in Figure 7. The results confirm that the crystallographic orientation is highly sensitive to the variation of deposition parameters. The texture coefficient, *TC_hkl_*, is calculated by using the following Harris formula [32].

(8) $$TC_{hkl}=\frac{I_{hkl}^{i}/I_{hkl}^{0}}{1/N\times \sum_{j=1}^{N}\left(I_{hkl}^{j}/I_{hkl}^{0}\right)},$$

Equation (8) describes the analysis of the relative peak intensities dependent on *I_hkl_*, i.e., the intensities observed from (*hkl*) lattice planes of the sample, and $I_{hkl}^{0}$ denotes the intensities of a standard Co powder. In this study, the intensity ratios of $I_{100}^{0},\;I_{002}^{0},\;and\;I_{101}^{0}$ were 26.6%, 27.8%, and 100%, respectively. *N* is the number of diffraction planes considered for the determination of *TC_hkl_*.

**Figure 8:**
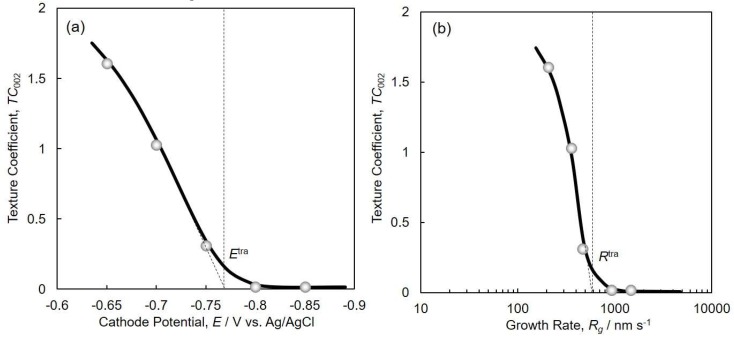
Effect of cathode potential (**a**) and growth rate (**b**) on the texture coefficient *TC*_002_ of electrodeposited Co nanocrystal arrays.

Figure 8 shows the effect of cathode potential (a) and growth rate (b) on the texture coefficient *TC*_002_ of the electrodeposited Co nanocrystal arrays. *TC*_002_ increased when the overpotential was decreased by shifting the cathode potential nobler than *E*^tra^ (ca. −0.77 V), and it reached a value of more than 1.5 at the cathode potential of ca. −0.65 V, as shown in Figure 8a. In a similar way, *TC*_002_ increased sharply when decreasing the growth rate of the Co nanocrystals to below the transition growth rate, *R*^tra^ (ca. 600 nm·s^−1^), and reached a value of more than 1.5 at the growth rate of ca. 200 nm·s^−1^, as shown in Figure 8b. Hence, these results reveal that c-axis (002) of hcp-Co is parallel oriented to the long axis of the Co nanocrystals at a cathode potential range nobler than *E*^tra^ or at a growth rate range smaller than *R*^tra^. The experimental results shown in Figure 8 are in good agreement with the theoretical estimation, as shown in Figure 2.

### 4.4. Perpendicular Magnetization of hcp-Co Nanocrystal Arrays

**Figure 9:**
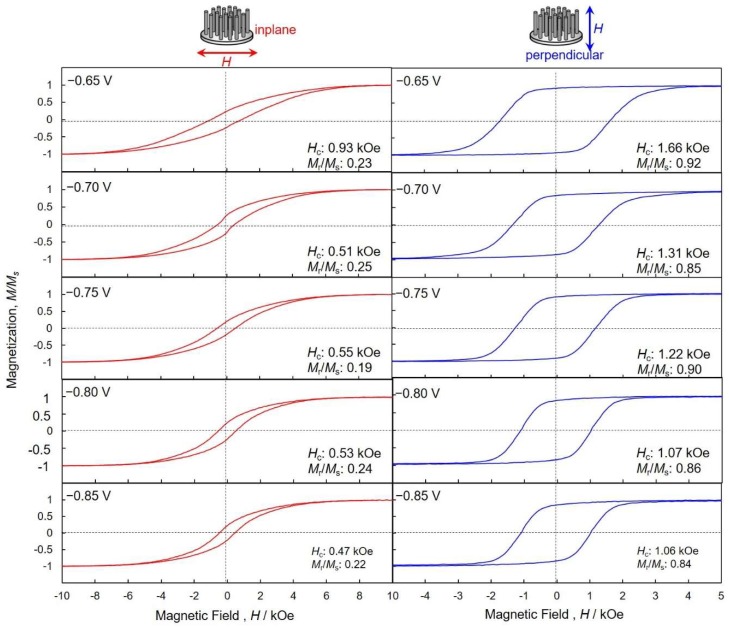
Magnetic hysteresis loops of Co nanocrystal arrays that were electrodeposited at each cathode potential ranging from −0.85 V to −0.65 V in the AAO nanochannel films. Magnetic field was applied to the in-plane (red lines) and perpendicular (blue lines) directions, with respect to the films.

Figure 9 shows magnetic hysteresis loops of the hcp-Co nanocrystal arrays that were electrodeposited at each cathode potential ranging from −0.85 V to −0.65 V in the AAO nanochannel films. Magnetic field was applied to the in-plane and perpendicular directions, with respect to the films. The in-plane direction corresponds to the short axis of a cylindrical Co nanocrystal, while the perpendicular direction corresponds to the long-axis of a cylindrical Co nanocrystal, as shown in the insets of Figure 9. The AAO nanochannel films with hcp-Co nanocrystal arrays were hardly magnetized in the inplane direction. Coercivity, *H*_c_, and squareness, *S* (*S* = *M*_r_/*M*_s_, the ratio of the remanent, *M*_r_, to the saturated magnetization, *M*_s_), of the hcp-Co nanocrystal arrays, that were electrodeposited at the cathode potential ranging from −0.85 V to −0.70 V, were ca. 0.51 kOe and ca. 0.22, respectively. This small squareness is strongly caused by the large demagnetization field, which is induced from the shape anisotropy of the Co nanocrystals. On the other hand, the coercivity of the nanocrystals that were electrodeposited at −0.65 V increased up to 0.93 kOe. This enhancement in the magnetic property is due to the increase of *TC*_002_, as shown in Figure 8. On the contrary, the nanocomposite films were easily magnetized in the perpendicular direction. This uniaxial magnetization behavior is caused by the large shape anisotropy of the Co nanocrystals and the magnetocrystalline anisotropy of hcp-Co. Coercivity and squareness of the Co nanocrystal arrays that were electrodeposited at −0.85 V were 1.06 kOe and 0.84, respectively. Furthermore, at the cathode potential of −0.65 V, those increased to as high as 1.66 kOe and 0.92, respectively. This result is in good agreement with the c-axis orientation data that were obtained from the XRD patterns (Figure 7). Hence, the long axis direction of the Co nanocrystal arrays corresponds well to the c-axis direction, which is magnetized more easily than the other directions.

**Figure 10:**
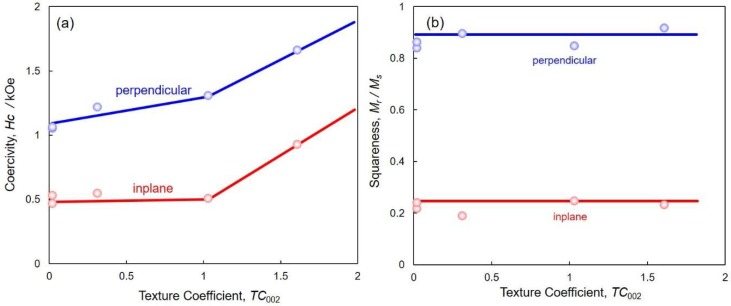
Effect of the texture coefficient on coercivity (**a**) and squareness (**b**) of electrodeposited Co nanocrystal arrays. Magnetic field was applied to the in-plane (red lines) and perpendicular (blue lines) directions, with respect to the films.

Figure 10 shows the effect of the texture coefficient on coercivity (a) and squareness (b) of the electrodeposited Co nanocrystal arrays. Increasing *TC*_002_ up to ca. 1.6 caused coercivity and squareness to increase to as high as 1.66 kOe and 0.92, respectively. Hence, these results reveal that the perpendicular magnetization performance is enhanced by increasing *TC*_002_, which results in the hcp-Co preferential crystal orientation of c-axis (002) to the long axis of the Co nanocrystals. This preferred (002) orientation texture, which is observed in Co nanocrystals that are electrodeposited at cathode potentials ranging from −0.75 to −0.65 V, results in an increase of the coercivity perpendicular to the long axis, as shown in Figure 10a. Kac et al. reported that Co nanocrystal arrays were able to be electrodeposited in nanochannels of a polycarbonate membrane film by using a potentiostatic electrodeposition technique [33]. They revealed that the squareness and coercivity of Co nanocrystal arrays (average diameter of 30 nm, average length of 1.5 µm, and average aspect ratio of ca. 50) were 0.72 and 0.85 kOe, respectively. Ren et al. also reported that Co nanocrystal arrays were able to be electrodeposited in nanochannels of an AAO membrane film by using an AC (200 Hz) electrodeposition technique [34]. They found that the squareness and coercivity of Co nanocrystal arrays (average diameter of 20 nm, average length of 5 µm and average aspect ratio of ca. 250) were 0.65 and 1.36 kOe. Han et al. also reported that hcp-Co nanocrystal arrays were able to be electrodeposited in nanochannels of an AAO membrane film by using an AC (200 Hz) electrodeposition technique [35]. They revealed that the squareness and coercivity of Co nanocrystal arrays (average diameter of 50 nm) were 0.69 and 1.57 kOe, respectively. Consequently, the squareness and coercivities of the Co nanocrystals arrays synthesized in this work are larger than those reported in other research [17,18,19,33,34,35]. This would be caused by the extremely large aspect ratio, which enhances the magneto-shape anisotropy to the long axis of hcp-Co nanocrystal arrays.

## 5. Conclusions

The formation work of a two-dimensional nucleus, *W_hkl_*, was estimated by Pangarov’s theory. According to our estimation, the *W*_002_ is smaller than *W*_100_ in the cathode potential range nobler than the transition potential, *E*^tra^ (ca. −0.77 V). Based on our experimental results, it has been revealed that *TC*_002_ increases when decreasing the overpotential by shifting the cathode potential nobler than *E*^tra^, and it reaches a value of more than 1.5 at the cathode potential of ca. −0.65 V. Hence, it has been found that c-axis (002) of hcp-Co is parallel oriented to the long axis of the Co nanocrystals at a cathode potential range nobler than *E*^tra^. Magnetic hysteresis loops in perpendicular directions to the AAO nanochannel film showed a strong magnetic anisotropy because of the high aspect ratios (ca. 1800) of all considered Co nanocrystal arrays. Therefore, the crystalline orientation and the shape anisotropy are the most important factors controlling the magnetic properties. The coercivity obtained in the magnetic field for the long axis direction of Co nanocrystal arrays with a preferred (002) orientation was 1.66 kOe. In contrast, the squareness obtained from Co nanocrystal arrays with a preferred (002) orientation increased up to 0.92 from 0.84 with an increase in *TC*_002_. This study illustrates the feasibility of improving the magnetic properties of Co nanocrystal arrays by controlling the degree of overpotential during electrodeposition.
